# Immunological imprinting of the antibody response in COVID-19 patients

**DOI:** 10.1038/s41467-021-23977-1

**Published:** 2021-06-18

**Authors:** Teresa Aydillo, Alexander Rombauts, Daniel Stadlbauer, Sadaf Aslam, Gabriela Abelenda-Alonso, Alba Escalera, Fatima Amanat, Kaijun Jiang, Florian Krammer, Jordi Carratala, Adolfo García-Sastre

**Affiliations:** 1 Department of Microbiology, Icahn School of Medicine at Mount Sinai, New York, NY USA; 2 Global Health and Emerging Pathogens Institute, Icahn School of Medicine at Mount Sinai, New York, NY USA; 3 Department of Infectious Diseases, Bellvitge University Hospital, Bellvitge Biomedical Research Institute (IDIBELL), University of Barcelona, L’Hospitalet de Llobregat, Barcelona, Spain; 4 Spanish Network for Research on Infectious Diseases (REIPI, RD16/0016, Carlos III Health Institute, Madrid, Spain; 5 Graduate School of Biomedical Sciences, Icahn School of Medicine at Mount Sinai, New York, NY USA; 6 Department of Pathology, Icahn School of Medicine at Mount Sinai, New York, NY USA; 7 Division of Infectious Disease, Department of Medicine, Icahn School of Medicine at Mount Sinai, New York, NY USA; 8 The Tisch Cancer Institute, Icahn School of Medicine at Mount Sinai, New York, NY USA

**Keywords:** Immunological memory, Humoral immunity, Antimicrobial responses, Viral infection

## Abstract

In addition to severe acute respiratory syndrome coronavirus 2 (SARS-CoV-2), humans are also susceptible to six other coronaviruses, for which consecutive exposures to antigenically related and divergent seasonal coronaviruses are frequent. Despite the prevalence of COVID-19 pandemic and ongoing research, the nature of the antibody response against severe acute respiratory syndrome coronavirus 2 (SARS-CoV-2) is unclear. Here we longitudinally profile the early humoral immune response against SARS-CoV-2 in hospitalized coronavirus disease 2019 (COVID-19) patients and quantify levels of pre-existing immunity to OC43, HKU1 and 229E seasonal coronaviruses, and find a strong back-boosting effect to conserved but not variable regions of OC43 and HKU1 betacoronaviruses spike protein. However, such antibody memory boost to human coronaviruses negatively correlates with the induction of IgG and IgM against SARS-CoV-2 spike and nucleocapsid protein. Our findings thus provide evidence of immunological imprinting by previous seasonal coronavirus infections that can potentially modulate the antibody profile to SARS-CoV-2 infection.

## Abstract (web summary)

In addition to SARS-CoV-2, other coronaviruses also infect human, but whether consecutive infections cross-modulate the induced immune response is still unclear. Here the authors show that SARS-CoV-2 infection boosts pre-existing responses to other coronaviruses, yet such back-boosting hampers the induction of specific antibodies against SARS-CoV-2.

## Introduction

Since January 2020, the Severe Acute Respiratory Syndrome Coronavirus 2 (SARS-CoV-2) virus has been spreading globally causing the first documented pandemic of coronavirus in history^1,2^. SARS-CoV-2 is a betacoronavirus that belongs to a large family of viruses capable to infect both mammals and birds. Humans are susceptible to at least other six viruses from the genus alpha and betacoronavirus^3^. All of them typically cause respiratory illness but to a different extent. While SARS-CoV-1 and Middle East Respiratory Syndrome Coronavirus, are highly pathogenic betacoronaviruses that have caused zoonotic outbreaks in humans in the last 20 years^4,5^, the alphacoronaviruses 229E and NL63, and the betacoronaviruses OC43 and HKU1, frequently cause mild upper respiratory tract disease and have been circulating in humans as seasonal viruses^3,6^. The ongoing pandemic of coronavirus disease 2019 (COVID-19), the disease caused by SARS-CoV-2, is still challenging healthcare systems and the research community. SARS-CoV-2 can cause a different range of clinical manifestations, from asymptomatic to severe respiratory syndrome. However, a high percentage of severe cases have been reported and estimated numbers of patients that succumbed to COVID-19 disease are more than 3 million according to WHO as May 2021^2,7^ (https://covid19.who.int/). Many vaccine candidates are being tested in clinical trials and several have already been authorized for use in the population^8–10^. However, we are still in an early phase and studies regarding vaccine effectiveness in special populations are needed. Similarly, longevity of the humoral immunity after infection and vaccination is still an ongoing debate.

One of the main targets of antibody responses to coronaviruses is the spike, the surface glycoprotein that mediates attachment to the host receptor and membrane fusion. Two subunits can be identified, the S1 subunit containing the receptor-binding domain (RBD), essential for binding to the entry receptor^11–13^; and the S2 subunit, responsible of virus cell fusion^14^. Different human coronaviruses use different domains to bind their human receptors and to mediate cell entry. While the human endemic betacoronaviruses OC43 and HKU1, bind to sialic acids, 229E alphacoronavirus uses human aminopeptidase N as a cellular determinant for susceptibility^15,16^. NL63, SARS-CoV, and SARS-CoV-2, in contrast, need direct interaction with the angiotensin-converting enzyme 2 to infect cells^13,17^. Therefore, antibodies directed against the RBD of human coronaviruses are capable to neutralize the virus^15,18,19^ and no cross-reactive neutralizing antibodies among seasonal human coronavirus are expected due to the high specificity of this process and the sequence divergence between the RBD of these viruses^20–23^. In addition, the more cross-reactive viral nucleoprotein (N) has also shown to be immunogenic and induce antibodies in COVID-19 patients. However, in contrast to RBD antibodies, N antibodies are not able to neutralize the virus in tissue culture^11,23,24^.

Several studies have demonstrated that T cells can recognize homologous epitopes shared between different endemic coronaviruses^25–30^. However, serum cross-reactivity between conserved epitopes from SARS-CoV-2 and seasonal human coronaviruses is still under investigation^23,31–33^ and the role of pre-existing humoral immunity and immunodominance for B cell responses needs to be addressed. Immune imprinting (or original antigenic sin), refers to the preference of the immune system to recall existing memory cells, rather than stimulating de novo responses when encountering a novel but closely related antigen^34^. This has been shown for viruses like influenza virus, in which subsequent infections with antigenically related strains produce a recall response or ‘back-boosting’ that generates an increase in antibody titers toward epitopes shared between the current and the historic strains encountered earlier in life^35–38^. Boost of cross-reactive antibody responses can also occur for viruses like dengue virus (DENV) upon secondary infections with a different serotype^39,40^. In this case, specific titers to the original DENV were higher than those specific to the second infecting DENV upon secondary DENV infection^41,42^.

Here, we profile the antibody responses of a longitudinal cohort of hospitalized patients with COVID-19. We characterize de novo antibody responses against SARS-CoV-2 and pre-existing immunity against selected endemic coronavirus being targeted by the humoral immune system to investigate the role of immunological imprinting on COVID-19 patients’ antibody response. We show that the induction of antibodies against conserved epitopes of seasonal coronaviruses may hinder the induction of specific antibodies toward divergent SARS-CoV-2 antigens. This study provides a dynamic characterization of the co-evolving nature of antibody responses to human coronaviruses, both seasonal and pandemic, and contributes to a better understanding of cross-reactive antibody responses and B cells immunodominance against human coronaviruses.

## Results

### The BACO cohort

Thirty-seven COVID-19 patients were recruited at the University Hospital of Bellvitge during the first wave of SARS-CoV-2 in Barcelona (Spain) from March 26, 2020 to May 28, 2020. Mean age was 65 years and 67% were male. Chronic comorbidities were frequent among COVID-19 patients (25, 67.7%). In particular, 16 (43.2%) of patients were obese (body mass index >30) at the time of hospitalization. A high percentage of patients had respiratory symptoms, such as coughing (26, 70.3%) and dyspnea (14, 37.8%), whereas diarrhea was also present in seven (18.9%) of the patients. While no remdesivir was available, lopinavir/ritonavir was used for 17 (45.9%) patients. All patients, except one (36, 97.3%), developed SARS-CoV-2 viral pneumonia and four (10.8%) required intensive care unit admission. Five (13.5%) patients died. Demographics, clinical characteristics, interventions, such as drug therapy and outcomes are detailed in Table 1.

**Table 1:** Demographics and clinical characteristics of the BACO cohort.

	Total (*n* = 37)
Demographics and comorbidities	
Age (mean, IQR)	67 (25)
Men (*n*, %)	25 (67.6)
Comorbidities (*n*, %)	25 (67.7)
Lung disease (*n*, %)	7 (18.9)
Diabetes mellitus	7 (18.9)
Heart disease (*n*, %)	5 (13.5)
Kidney disease (*n*, %)	3 (8.1)
Obesity (*n*, %)	16 (43.2)
SOTR (*n*, %)	1 (2.7)
Signs and symptoms	
Days from symptom onset to enrollment (mean, range)	7.19 (2–14)
Days of fever (mean, range)	4.68 (0–12)
Throat ache (*n*, %)	4 (10.8)
Cough (*n*, %)	26 (70.3)
Dyspnea (*n*, %)	14 (37.8)
Diarrhea (*n*, %)	7 (18.9)
Sp02 < 94% (*n*, %)	14 (37.8)
Drug therapy	
Hydroxychloroquine (*n*, %)	36 (97.3)
Lopinavir/Ritonavir (*n*, %)	17 (45.9)
Tocilizumab (*n*, %)	10 (27)
Antibiotics (*n*, %)	19 (51.4)
Corticosteroids (*n*, %)	18 (48.6)
Outcomes	
Pneumonia (*n*, %)	36 (97.3)
ICU (*n*, %)	4 (10.8)
Days from hospitalization to ICU (mean, range)	9.5 (5–12)
Days in ICU (mean, range)	15 (15–22)
Non-mechanical ventilation (*n*, %)	11 (29.7)
Mechanical ventilation (*n*, %)	2 (5.4)
Nosocomial co-infection (*n*, %)	2 (5.4)
Mortality (*n*, %)	5 (13.5)
Days of hospitalization (mean, range)	11.2 (2–47)

*SOTR* solid organ transplant recipient, *SpO2* *<* *94%* pulse oximetry below 94%, *ICU* intensive care unit.

Acute blood samples were collected longitudinally in the BACO cohort at the recruitment upon hospital admission, and at days 3 and 7 in 33 (89.1%) and 22 (59.4%) patients, respectively. Mean time from symptom onset to inclusion in the study was 7 days (range 2–14). Most of the patients (25, 67.5%) were recruited within the first week of symptom onset, whereas 12 (32.4%) patients had longer periods until hospitalization. COVID-19 survivors were followed up in the convalescence period and 28 out of 32 survivors (87.5%) had another blood draw after hospital discharge with a mean time of 46 days post recruitment (range, 30–56 days).

### COVID-19 patients developed anti-SARS-CoV-2 antibodies linked to back-boosting of antibodies against S2 domain of betacoronaviruses

**Fig. 1:**
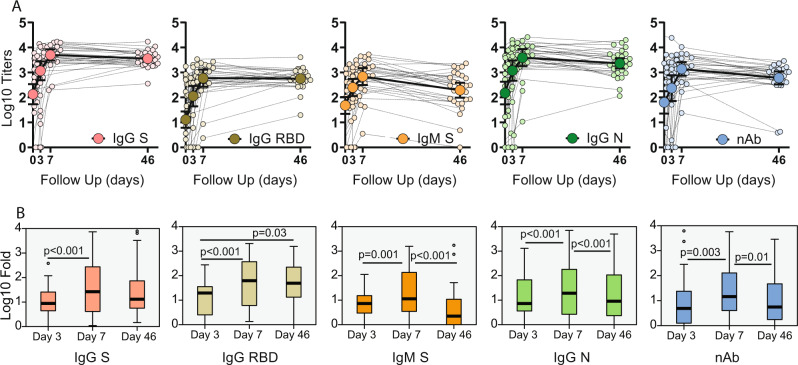
Longitudinal antibody response to SARS-CoV-2 antigens. Serum from hospitalized COVID-19 patients was analyzed at baseline, at hospital recruitment and days 3 and 7. A subsequent sample was collected in the convalescence period in the COVID-19 survivors with mean time of 46 days. **A** Longitudinal profile of antibodies against SARS-CoV-2. Antibody titer was quantified as area under the curve (AUC) after serial serum dilution for each sample (Supplementary Fig. 1). Calculated AUC at each time is shown to quantify changes over time for each individual (small dots) against immunoglobulin G (IgG) spike, IgG receptor-binding domain (RBD), immunoglobulin M (IgM) spike and IgG nucleocapsid (N); and neutralizing activity (nAb) as inhibitory concentration 50% (IC50%). Geometric mean titer (GMT, big dots) and confidence interval (CI 95%) are also shown. **B** Boxplot diagram of geometric mean fold rise (GMFR) antibody titers against SARS-CoV-2 at the same time points: IgG spike, IgG RBD, IgM spike and IgG NP; and neutralizing activity (nAb). Related-samples Friedman’s two-way ANOVA was performed. Significant adjusted *p* values after pairwise comparisons are shown for each comparison. Black bar indicates GMFR values, box indicates IQR (Q1–Q3), lines indicate minimum and maximum. Outliers from the observed distribution are shown. Total *n* = 116 biologically independent serum samples (day 0 = 37, day 3 = 29, day 7 = 22, day 46 = 28). *n* = 116 biological samples examined against four different SARS-CoV-2 substrates for ELISA assays; ELISAs for each substrate were run once each. *N* = 116 serum samples examined over two independent experiments for neutralization assays.

To profile the early antibody response in COVID-19 patients, we investigated the levels of neutralizing antibodies against authentic SARS-CoV-2 virus and IgG/IgM ELISAs against multiple antigens including the full-length spike (S), the spike RBD S and the N of SARS-CoV-2. IgG and IgM levels were quantified as area under the curve (AUC) by plotting normalized optical density (OD) values against the reciprocal serum sample dilutions for ELISAs (Supplementary Fig. 1A). To improve visualization, the longitudinal antibody profile of each individual patient together with the geometric mean titer (GMT, CI 95%) at each time point is shown for AUC ELISA and neutralizing titers in Fig. 1A and Supplementary Table 1. All patients developed detectable levels of neutralizing antibodies at day 7 post recruitment while levels remained stable during the convalescent phase, except for two survivors. Similar responses were found by ELISA, although higher levels of antibodies against IgG S compared to IgG RBD were present. When comparing to the induction of anti-spike antibodies, the IgG isotype reached higher titers than the IgM isotype, whereas anti-N protein IgG had similar induction than the anti-S IgG. We then determined fold increase of antibody titers from baseline levels. Overall, all patients had a high induction of SARS-CoV-2 S and RBD antibodies at day 7 post recruitment. IgG titers against the S and RBD of SARS-CoV-2 remained stable at the convalescent time point with similar levels compared to peak titers at day 7. By contrast, IgM against the S, IgG against N and neutralizing titers against authentic SARS-CoV-2 virus decreased to levels resembling those at day 3. Geometric mean fold rise (GMFR) and adjusted *p* values on pairwise comparisons after related samples Friedman’s two-way ANOVA at each time points are shown Fig. 1B. We next tested the correlation between neutralization activity and levels of anti-SARS-CoV-2 antibodies. Scatterplot matrices shown in Supplementary Fig. 2 indicate that the antibodies detected against SARS-CoV-2 antigens correlated well with neutralizing activity, with Pearson R^2^ ranging from 69 to 81% in the case of IgG against the RBD S of SARS-CoV-2.

**Fig. 2:**
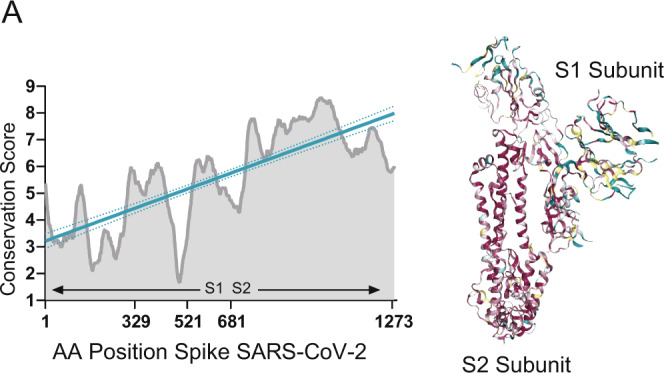
Conservation of SARS-CoV-2 S protein. **A** Multiple sequence alignment was generated by the ConSurf algorithm (https://consurf.tau.ac.il) using the chain A of the SARS-CoV-2 spike protein in the closed state (PDB ID 6VXX) as a reference. Amino acid conservation scores were classified into nine levels. Structure of the SARS-CoV-2 S protein (chain A) with amino acid residues colored according to conservation on a scale from green (1, most variable) to dark purple (9, most conserved) is also shown.

**Fig. 3:**
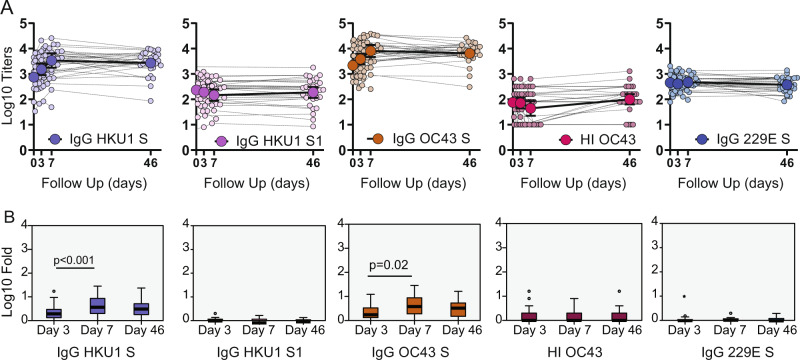
Longitudinal antibody response to selected seasonal human coronaviruses antigens. Serum from hospitalized COVID-19 patients was analyzed at baseline, at hospital recruitment and days 3 and 7. A subsequent sample was collected in the convalescence period in the COVID-19 survivors with mean time of 46 days. **A** Longitudinal profile of antibodies against betacoronavirus (HKU1 and OC43) and alphacoronavirus (229E) antigens. Antibody titer was quantified as area under the curve (AUC) after serial serum dilution for each sample (Supplementary Fig. 1). Calculated AUC at each time is shown to quantify changes over time for each individual (small dots) against immunoglobulin G (IgG) HKU1 spike, IgG HKU1 S1, IgG OC43 spike and IgG 229E. Geometric mean titer (GMT, big dots) and confidence interval (CI 95%) are also shown. Hemagglutination inhibition (HI) assay were also performed for OC43 and GMT of end point titers are shown at each time point. **B** Boxplot diagram of geometric mean fold rise (GMFR) antibody titers against seasonal coronaviruses at the same time points: IgG HKU1 spike, IgG HKU1 S1, IgG OC43 spike and IgG 229E; and HI titer. Related-samples Friedman’s two-way ANOVA was performed. Significant adjusted *p* values after pairwise comparisons are shown for each comparison. Black bar indicates GMFR values, box indicates IQR (Q1–Q3), lines indicate minimum and maximum. Outliers from the observed distribution are shown. Total *n* = 116 biologically independent serum samples (day 0 = 37, day 3 = 29, day 7 = 22, day 46 = 28). *n* = 116 biological samples examined against four different seasonal coronavirus substrates for ELISA assays; ELISAs for each substrate were run once each. *N* = 116 serum samples examined over two independent experiments for hemagglutination assays.

The S gene of SARS-CoV-2 is highly divergent from human seasonal coronaviruses (hCoV). Infection with endemic hCoV in humans happens frequently^3,6,43^, causing mild respiratory disease. Multiple sequence alignment (MSA) between the S of SARS-CoV-2 and selected seasonal coronaviruses showed amino acid identity ranging from 28% for alphacoronaviruses (229E) and 32.5% and 33% for betacoronaviruses (OC43 and HKU1, respectively). To identify conserved amino acid regions, we also estimated the relative conservation scores of the S protein of SARS-CoV-2 using the chain A of the SARS-CoV-2 spike protein in the closed state as a reference. MSA and relative amino acid conservation was determined by using the ConSurf server. Figure 2A shows the conservation score for each amino acid position and projected on the S protein structure. Evolutionary conservation analysis showed that the S2 subunit had the highest degree of identity among the sequences tested. Given the high probability of previous exposure to seasonal coronaviruses in the BACO cohort, we screened levels of antibodies against the spike of alphacoronavirus 229E and betacoronaviruses HKU1, OC43. Antigens tested included full-length S protein for all three endemic coronaviruses together with the less conserved HKU1 S1 subunit (Supplementary Fig. 3A). Remarkably, COVID-19 patients exhibited an outstanding back-boosting of antibodies to the beta- CoV spikes tested, with similar a longitudinal profile as the one observed for the SARS-CoV-2 spike and for SARS-CoV-2 neutralizing titers (Fig. 3A). The back-boost was higher at day 7, with a GMFR from baseline levels of 3.8 and 4 for HKU1 S and OC43 S, respectively (Fig. 3B, Supplementary Table 1). While IgG levels against 229E were already high at baseline, no increase was detected at any time point during the follow-up on patients with COVID-19. Interesting, no back-boosting was found when we tested antibody titers against the more divergent S1 subunit of HKU1, pointing to an increase of immune responses towards conserved epitopes of the S2 subunit of the spike protein of beta-human coronaviruses. Similar to influenza viruses, HKU1 and OC43 use sialic acids as canonical receptor to infect human cells^16^. This is mediated by an additional surface protein in these viruses with hemagglutination (HA) activity (hemagglutinin-esterase (HE) protein). No increase in OC43 HA inhibitory antibodies was found in COVID-19 patients, consistent with the lack of HE in SARS-CoV-2. Longitudinal profile and fold increase antibody titers to selected seasonal human coronaviruses antigens are shown in Fig. 3 and Supplementary Table 1.

**Fig. 4:**
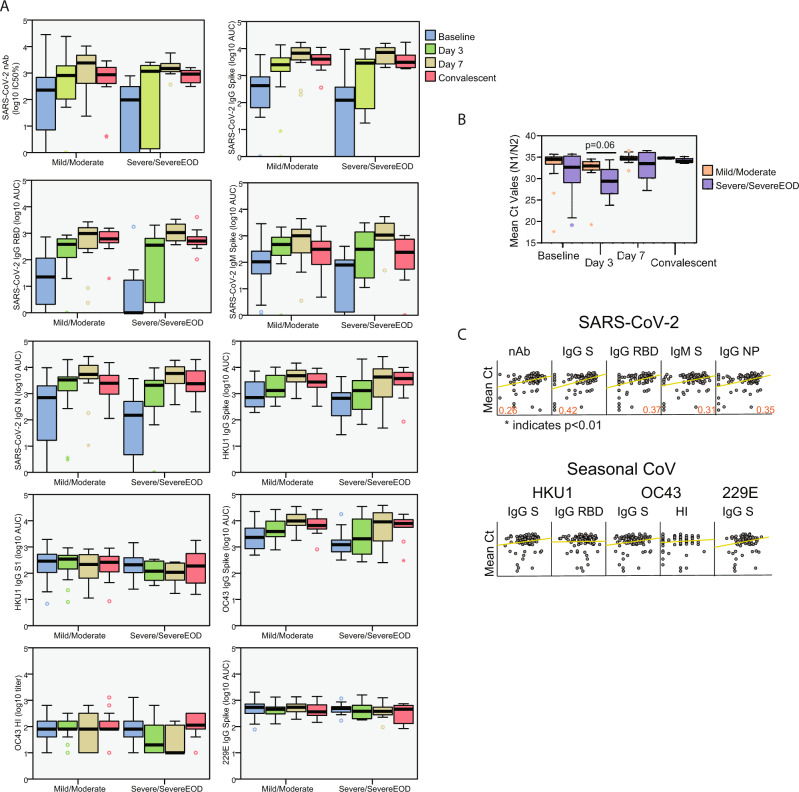
Antibody response according to disease severity and viral loads in the BACO cohort. **A** Boxplot diagram of ELISA as area under the curve (AUC) titers against SARS-CoV-2 and endemic human coronaviruses at each time point in mild/moderate vs. severe COVID-19: IgG spike, IgG RBD, IgM spike and IgG NP; and neutralizing titer (IC50%); and HKU1 IgG spike, HKU1 IgG S1 subunit, OC43 IgG spike and 229E IgG spike; and OC43 hemagglutination titers. Black bar indicated median values, box indicates IQR (Q1–Q3), and lines indicate minimum and maximum. Outliers from the observed distribution are shown when present in each case. Total *n* = 116 biologically independent serum samples (day 0 = 37, day 3 = 29, day 7 = 22, day 46 = 28). *n* = 116 biological samples examined against eight different SARS-CoV-2 and seasonal coronavirus substrates for ELISA assays; ELISAs for each substrate were run once each. *N* = 116 serum samples examined over two independent experiments for neutralization and hemagglutination assays. **B** Boxplot diagram of mean threshold cycle (Ct) values in mild/moderate vs. severe COVID-19 during the follow-up. N protein was detected by RT-qPCR. Black bars indicate median values, the box indicates IQR (Q1–Q3), and lines indicate minimum and maximum. Outliers from the observed distribution are shown when present in each case. Total *n* = 93 biologically independent nasopharyngeal swab (day 0 = 37, day 3 = 28, day 7 = 22, day 46 = 6). *n* = 93 biological samples examined against two different SARS-CoV-2 primers over two independent experiments each. Mann–Whitney *U* test for independent samples was performed. Reported *p* values are based on two‐tailed tests. **C** Scatterplot of the relationship between measured SARS-CoV-2 and seasonal coronaviruses antibody responses and Ct values in the COVID-19 patients. Pearson coefficient of statistically significant correlations is indicated in red. Matrix axis are log10 values scaled from 0 to 4. Total *n* = 93 biologically independent nasopharyngeal (NP) swab and 93 paired serum samples. Pearson correlation was calculated based on matched NP and serum samples. *P* values for statistically significant values are shown and based on two‐tailed tests. Source data are provided as a Source Data File.

To test whether the antibody response characterized in the BACO cohort correlated with disease trajectory, we grouped patients according to disease phenotype. Patients were assigned as mild/moderate (*N* = 26, 70.3%) or severe/severe end-of organ disease (EOD, *N* = 11, 29.7%) based on a previously described severity scale^44^. No statistically significant differences were found between humoral immune response in patients with mild and severe/severe EOD disease, but the latter tended to have a delay in the antibody response towards SARS-CoV-2 antigens compared to moderate cases (Fig. 4A). Patients with severe disease had lower Ct values, and therefore higher viral loads (Fig. 4B). Besides, a positive correlation was found between anti- SARS-CoV2 antibodies and mean Ct values in paired nasopharyngeal swabs of COVID-19 patients acknowledging an interplay between antibodies and virus control and disease severity in COVID-19 patients. However, no correlation was found between antibodies against seasonal coronaviruses and viral loads in the BACO Cohort (Fig. 4C).

### Immunological imprinting results in a bias in the induction of antibodies to conserved vs. variable regions of the SARS-COV-2 spike

Given the strong back-boosting observed to the conserved epitopes of the S domains of human betacoronaviruses in patients with COVID-19, we next investigated whether a strong back-boosting might reduce the induction of de novo humoral immune responses against specific epitopes of the spike of SARS-CoV-2 defined as fold induction over baseline levels.

**Fig. 5:**
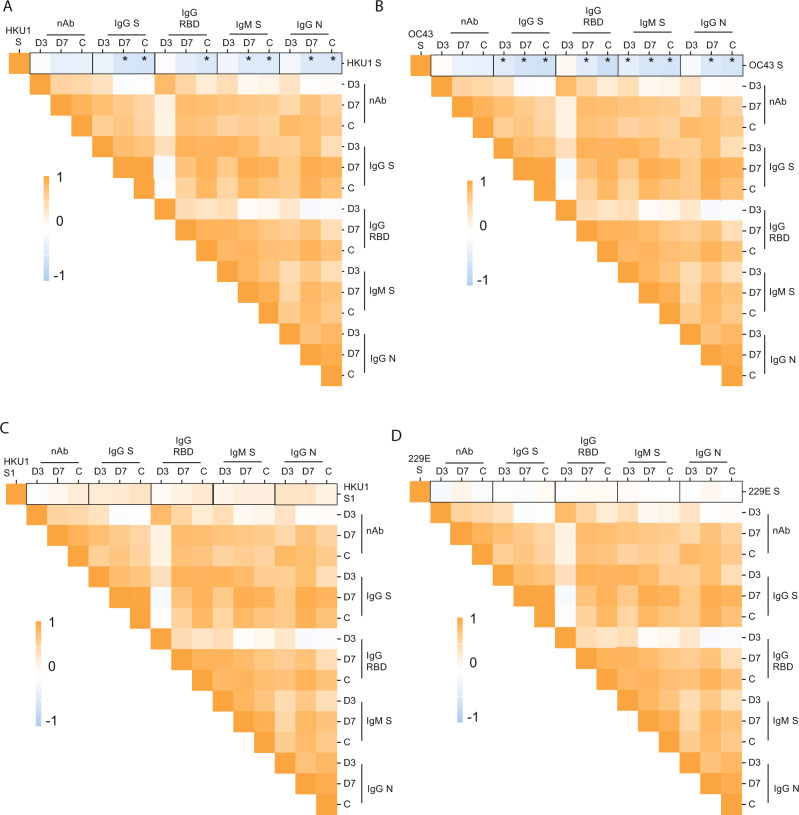
Immunological imprinting on SARS-CoV-2 antibody response. **A**, **B** Heat map of Pearson correlation matrices between pre-existing levels of seasonal hCoV (A IgG HKU1 S; and B IgG OC43 S) and fold induction of SARS-CoV-2 antibodies at each time point: neutralizing (nAb), IgG spike, IgG RBD, IgM spike and IgG NP. Statistically significant correlations in the underlined intersections are indicated with asterisk (*); D3: day 3; D7: day 7; C: convalescence. **C**, **D** Scatterplot of baseline IgG levels for HKU1 and OC43 S protein and fold induction of SARS-CoV-2 antibodies: neutralizing (nAb), IgG spike, IgG RBD. Overlay shows relationship with induction of de novo antibodies against SARS-CoV-2 at each time point. Fitted linear regression and standardized beta coefficient (95% confidence interval, CI) for significant linear regressions are shown.

**Fig. 6:**
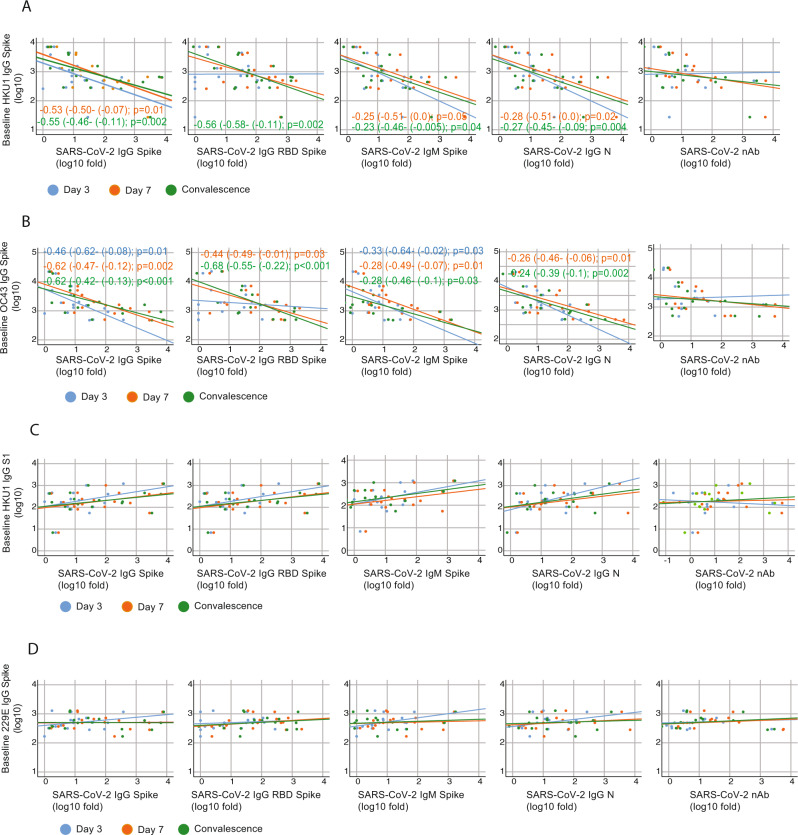
Cross-reactivity with conserved epitopes against selected betacoronaviruses predicts negative influence on de novo anti-SARS-CoV-2 antibody responses. **A**–**D** Scatterplot of baseline IgG levels for HKU1, OC43 and 229E S protein; and HKU1 S1 and fold induction of SARS-CoV-2 antibodies: neutralizing (nAb), IgG spike, IgG RBD, IgM spike, and nucleoprotein. Overlay shows relationship with induction of de novo antibodies against SARS-CoV-2 at each time point. Fitted linear regression and standardized beta coefficient (95% Confidence Interval, CI) for significant linear regressions are shown. Reported *p* values are based on two‐tailed tests.

**Fig. 7:**
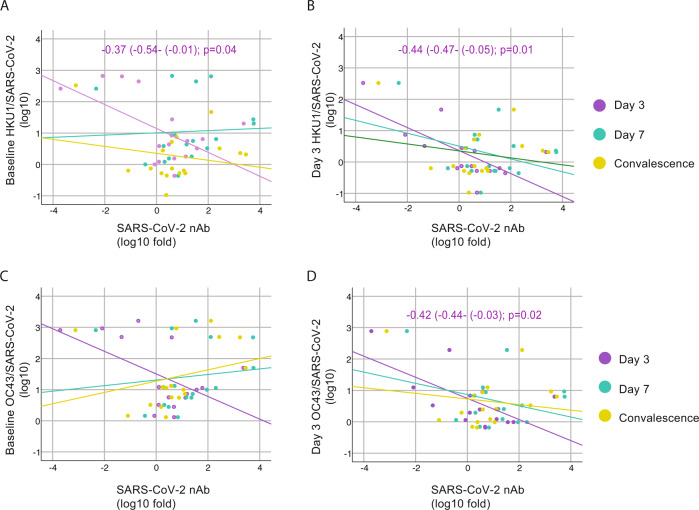
Influence of levels of back-boosting to HKU1 and OC43 normalized by levels of anti- SARS-CoV-2 antibodies on neutralizing antibodies induction. **A**–**D** Scatterplot of baseline and day 3 IgG levels for HKU1 and OC43 S protein normalized by the levels of SARS-CoV-2 IgG and their relationships with fold induction of SARS-CoV-2 neutralizing antibodies (nAb) over time. Overlay shows linear regression at each time point. Fitted linear regression and standardized beta coefficient (95% confidence interval, CI) for significant regression are shown. Reported *p* values are based on two‐tailed tests.

To test this hypothesis, we examined the relationship between pre-exposure to HKU1, OC43, and 229E viruses and the induction of SARS-CoV-2 S, RBD, and N antibodies in our cohort, and determined Pearson correlation coefficients between IgG levels at baseline against seasonal human coronaviruses and the fold induction of SARS-CoV-2 antigens at days 3, 7 and convalescence. Pearson correlation matrices according to seasonal coronavirus subtype are shown in Fig. 5. Striking differences were found according to virus types. While pre-existing IgG levels against HKU1 and OC43 spike protein negatively impacted the induction of de novo IgG and IgM against SARS-CoV-2 antigens, including S and N protein (Fig. 5A, B), no influence was found when testing the relationship between pre-existing anti-229E spike IgG levels (Fig. 5D). Moreover, correlations became stronger over time, and while this correlation was lower at day 3, a stronger correlation was found at day 7, and convalescence time points in the surviving patients. Besides, a comparable performance was observed when testing the subsequent induction of the IgG antibodies against the variable RBD domain of SARS-CoV-2 spike. This result suggests that pre-existing immunity against seasonal betacoronaviruses biases the humoral response towards betacoronaviruses cross-reactive antibodies in detriment of antibodies against the more divergent and antigenically unique domains of the S of SARS-CoV-2, such as those of the RBD domain (Fig. 5A, B). This was also evidenced by the lack of impact of pre-existing HKU1 S1 IgG levels (S1 is divergent and harbors the RBD) on specific SARS-CoV-2 antibodies induction (Fig. 5C). Thus, only the levels of antibodies against cross-reactive epitopes of human betacoronaviruses had an effect on the subsequent antibody response to SARS-CoV-2 unique spike antigens. Because neutralization activity has been linked to in vivo protection after challenge with SARS-CoV-2^45^, we also tested if immune imprinting could hinder the induction of neutralizing antibodies against SARS-CoV-2. No significant correlation was found. However, linear regression analysis determined a standardized beta coefficient of −0.32 (95% CI −0.35–0.05, *p* = 0.13) and −0.31 (95% CI −0.28–0.02, *p* = 0.1) at day 7 and convalescence time points, respectively, for pre-existing HKU1 spike antibody levels approximating a negative impact of HKU1 pre-existing immunity on induction of neutralizing antibodies against SARS-CoV-2 in COVID-19 patients over time (Fig. 6A). A similar trend was found for the levels of pre-existing antibodies against the OC43 spike (Fig. 6B). Interestingly, the impact of back-boosting on IgM against the S protein was smaller when compared to IgG S or RBD. Scatterplots and the predicted regression lines for the relationship of induction of antibodies against SARS-CoV-2 and pre-exposure to betacoronaviruses are shown in Fig. 5A–D according to time points in the longitudinal follow-up. To assess neutralization potency according to the levels of pre-existing levels of seasonal coronaviruses, we normalized levels of IgG against seasonal human coronavirus antigens by the levels of anti- spike IgG from SARS-CoV-2 virus at the same time points. This analysis allows for comparison of high vs. low presence of pre-existing antibodies toward OC43, 229E, or HKU1 (antibodies at baseline) against the elicitation of SARS-CoV-2 antibodies over the observation course. We then tested whether those patients with higher hCoV/SARS-CoV-2 IgG ratio had lower induction of neutralizing antibodies. After linear regression analysis some disparities were found (Fig. 7). In general, the higher hCoV/SARS-CoV-2 IgG ratio for HKU1 and OC43 IgG S at baseline and day 3, the lower was the induction of antibodies with neutralizing activity to SARS-CoV-2, suggesting some limitations for the ability to elicit robust protective antibody responses against novel antigenic epitopes of SARS-CoV-2 in patients with high levels of cross-reactive antibodies against circulating betacoronaviruses.

**Fig. 8:**
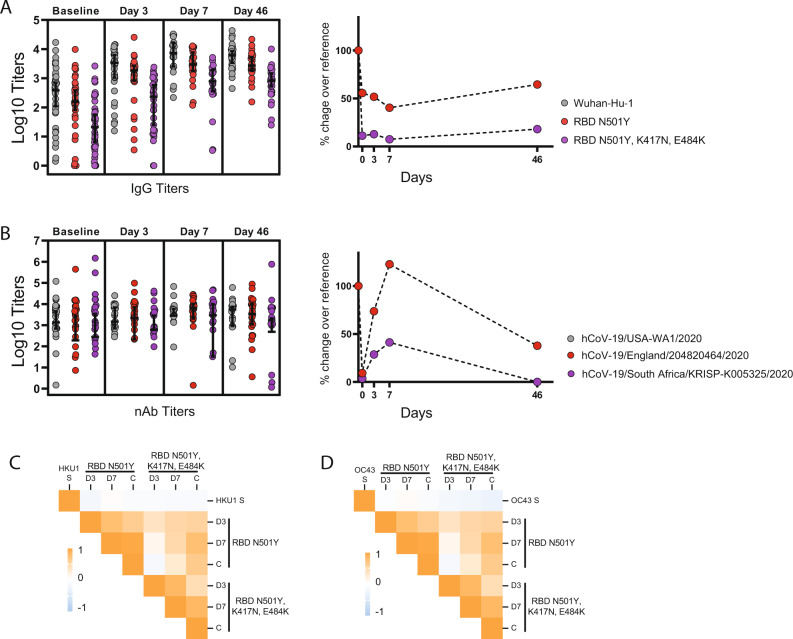
Imprinting and antibody response against emerging variants of SARS-CoV-2. **A** ELISA against the receptor-binding domains (RBDs) of Wuhan-Hu-1 (reference), and mutated RBDs representative of UK (N501Y) and South African (K417N, E484K and N501Y) variants. **B** Neutralizing titers against the authentic hCoV-19/England/204820464/2020 (B.1.1.7) and hCoV-19/South Africa/KRISP-K005325/2020 (B.1.351). Errors indicate geometric mean titer (GMT) and confidence interval (CI 95%) at each time point for ELISA against each RBD or neutralizing titers against each variant. Percentage of decrease titers compared to reference has been calculated and data are shown in the right for ELISA and nAb titers. Total *n* = 116 biologically independent serum samples (day 0 = 37, day 3 = 29, day 7 = 22, day 46 = 28). *n* = 116 biological samples examined against three different SARS-CoV-2 RBDs; ELISAs for each substrate were run once each. *N* = 116 serum samples examined for three different SARS-CoV-2 variants over two independent experiments each. **C**–**D** Heat map of Pearson correlation matrices between pre-existing levels of seasonal CoVs: IgG HKU1 S; and B IgG OC43 S; and fold induction of antibodies against RBD N501Y and RBD N501Y, K417N, E484K RBD at each time point. D3 day 3, D7 day 7, C convalescence.

Finally, and to test whether imprinting on B cell compartment could also influence antibody responses against more divergent mutated spike proteins from SARS-CoV-2 variants, we measured antibody responses against the spike protein of two SARS-CoV-2 variants. These variants, B.1.1.7 and B.1.351 emerged in late 2020 in United Kingdom and South Africa, respectively. Both B.1.1.7 and B.1.351 bear a N501Y mutation within the RBD while B.1.351 contains also K417N, E484K changes. In addition, further mutations can be found outside of the RBD domain. We performed ELISA against the B.1.1.7 and B.1.351 RBDs as well as neutralization assays against the authentic hCoV-19/England/204820464/2020 (B.1.1.7) and hCoV-19/South Africa/KRISP-K005325/2020 (B.1.351) variants. Interesting, when percentage of decrease compared to the reference was calculated, we found that responses targeting the RBD dropped from 50 to almost 100% for B.1.1.7 and B.1.351, respectively (Fig. 8A). In contrast, neutralizing titers against B.1.1.7 were similar to USA-WA1/2020, while percentage of decrease respect to B.1.351 was around 50%, indicating presence of neutralizing antibodies directed against epitopes different to those contained in the RBD, such as those directed against the N-terminal domain (Fig. 8B). Finally, we calculated Pearson correlation coefficients to examine the relationship between seasonal coronavirus HKU1 and OC43 pre-existing immunity and the ELISA antibody responses against the mutated RBDs. Pearson correlation matrices in Fig. 8C, D shows the relationship between pre-existing antibody levels against OC43 and HKU1 and fold induction against RBDs containing N501Y only, or N501Y, K417N, and E484K mutations. No significant correlation was found between pre-exposure to seasonal coronaviruses and responses against the mutated RBDs. The BACO cohort presented in here was enrolled in the first wave of SARS-CoV-2 in Spain, and the likelihood of being infected against a similar variant to Wuhan-Hu-1 is high. It is likely that the drop on RBD titers for the variants is responsible for the lack of detection of an imprinting effect with these variants.

## Discussion

Our findings provide a dynamic characterization of the antibody response to SARS-CoV-2 in COVID-19 patients and provide evidence of immune imprinting in these patients. Our results demonstrate back-boosting in the BACO cohort against the conserved epitopes of the spike protein of OC43 and HKU1 betacoronaviruses. No induction was detected for the variable regions of these viruses, such as the S1 domain, or to more divergent seasonal alphacoronaviruses, such as 229E. Although antibody cross-reactivity has been reported in cross-sectional studies^22,23,25,32^, our cohort has allowed for quantification and detailed representation of the longitudinal outcome of the immune response by taking into consideration past exposure to related antigens. Neutralization activity of antibodies might be used as a proxy for protection against SARS-CoV-2 infection^46,47^. IgG responses to the spike and RBD of SARS-CoV-2 showed persistence over the time period of our study with slight changes in antibody levels in convalescent sera as compared to the peak of antibody induction at day 7. Importantly, immunity to other betacoronavirus spikes, like HKU1 and OC43, limited the induction of de novo responses to all SARS-CoV-2 antigens tested. All patients also developed detectable levels of spike IgG/IgM and N IgG. Although no significant correlation was found between pre-exposure to seasonal coronaviruses and induction of protective antibodies with neutralizing activity, simple linear regression estimated a negative relationship, and the predicted line approximated a negative influence on development of de novo neutralizing antibodies over time. Similarly, baseline antibody levels to HKU1 or OC43 spike after SARS-CoV-2 IgG levels normalization limited the induction of neutralizing antibody levels after in the follow-up.

While we could not find statistically significant differences for antibody levels in patients with mild vs. severe disease, the latter showed a delay in antibody responses. Moreover, anti-SARS-CoV-2 antibodies inversely correlated with viral loads in respiratory samples, whereas virus clearance could not be linked to back-boosting of antibodies toward the S2 subunit of the seasonal human coronaviruses. Importantly, several reports have shown cross-reactivity between pre-existing memory T cells to seasonal coronaviruses and SARS-CoV-2^25,48^ pointing to a potential role of heterologous immunity as an additional mechanism of protection or even differences on COVID-19 outcomes. However, our results allow for a contrasting hypothesis in which early priming of the memory B cell compartment due to pre-exposure to seasonal coronaviruses could dampen secondary responses toward new epitopes of SARS-CoV-2. Nonetheless, all patients from the BACO cohort developed antibody responses against SARS-CoV-2 antigens and specific neutralizing antibodies. In addition, SARS-CoV-2 is evolving, and some variants including 501Y spike mutations have emerged and rapidly spread in countries, such as UK, South Africa, and Brazil (https://www.who.int/csr/don/31-december-2020-sars-cov2-variants/en/). These variants contain mutations that introduce amino acid changes in RBD residues targeted by neutralizing antibodies and therefore have functional significance. There is a general concern on whether new emerging variants (also known as variants of concern, VOC) could evade immunity generated not only by previous infections but also vaccination causing a drop on the effectiveness of COVID-19 vaccines. It is possible that first-generation COVID-19 vaccines will need to be updated according to the circulating variants in the future.

Our observation has important impact of on the development of COVID-19 vaccines and the potential interactions with pre-existing immunity should be taken into consideration in the path to optimal vaccines. COVID-19 vaccines in use aim at the induction of responses against the full-length S protein of SARS-CoV-2^49^, which is known to contain cross-reactive non-neutralizing epitopes that are shared with seasonal human betacoronaviruses. A similar scenario to our studies in infected people could be proposed for the vaccines, with some differences due to the nature of the stimulus itself. Back-boost of cross-reactive antibody responses might lead to less protective antibodies directed against non-neutralizing conserved epitopes between the S antigen of the vaccine and the S proteins of seasonal human betacoronaviruses^50^. On the other hand, it is also possible that cross-reactive antibodies provide protection from severe disease outcomes by immune mechanisms of action different from those involved on in vitro virus neutralization, such as antibody-dependent cytotoxicity. That is the case for broadly cross-reactive and non-neutralizing anti-influenza antibodies targeting the conserved stalk domain of the hemagglutinin protein of influenza viruses. HA stalk antibodies can mediate antibody-dependent cell cytotoxicity, contributing to protection from disease severity independently of neutralizing activity^51^. Whether in vitro non-neutralizing anti-SARS-CoV-2 antibodies contribute to protection or disease or are neutral is still not clear.

Our study has several limitations. We comprehensively characterized antigen specificity, neutralization potency, and viral cross-reactivity against multiple coronaviruses over time. However, the number of subject enrolled remained relatively small due to the challenges and restrictions faced by the hospitals during the initial spread of SARS-CoV-2, underpowering the conclusions of this study. In addition, all the patients enrolled required hospitalization, and the pre-existing immunity of asymptomatic or mild cases of COVID-19 could not be characterized in this study. Still, our results demonstrate that the antibody response against SARS-CoV-2 infection and, potentially vaccination, is influenced by imprinting of the B cell compartment due to previous exposure to seasonal human betacoronaviruses. This is consistent with additional recent studies^33^. It will be important to investigate the potential functional consequences of this imprinting in the induction of protective immune responses after SARS-CoV-2 infection and vaccination in the long term, and in the very likely case that the current pandemic evolves into epidemic outbreaks.

## Methods

### Experimental model and subject details: The BACO cohort

An observational prospective human cohort study of COVID-19 was carried out during the first pandemic wave (March–May 2020) of SARS-CoV-2 in Barcelona (Spain) and was termed the BACO Cohort. A positive case was defined according to international guidelines when a nasopharyngeal (NP) swab tested positive for SARS-CoV-2 by reverse transcriptase quantitative polymerase chain reaction (RT-qPCR) upon hospital admission. All patients or their legally authorized representatives provided informed consent. Serum and samples were collected at the enrollment in the study (baseline), and at days 3 and 7 post enrollment. A convalescence sample was collected from survivors after recovery and hospital discharge with a mean time of 46 days (range, 30–56 days). The total number of serum samples was 116. Data on demographics, including age and sex, comorbidities, clinical signs and symptoms, interventions, and outcomes are described in Table 1. Severity of COVID-19 was assigned following a described severity scale based on oxygen saturation (SpO_2_), presence of pneumonia/imaging, oxygen support defined as use of high-flow nasal cannula (HFNC), non-rebreather mask (NRB), bilevel positive airway pressure (BIPAP) or mechanical ventilation (MV); and kidney (creatinine clearance, CrCl) and liver (alanine aminotransferase, ALT) function^44^: mild (SpO2 > 94% AND no pneumonia), moderate (SpO2 < 94% AND/OR pneumonia), severe (use of HFNC, NRB, BIPAP or MV AND no vasopressor use AND CrCl >30 AND ALT < 5x upper limit of normal) and severe with end-of organ disease (Use of HFNC, NRB, BIPAP or MV AND vasopressor use OR CrCl >30 or new HD OR ALT < 5x upper limit of normal).

The study protocol was approved by the Institutional Review Board of University Hospital of Bellvitge, Barcelona, Spain; and by the Icahn School of Medicine at Mount Sinai, New York, US.

### Cell lines

Vero E6 cells were originally purchased from the American Type Culture Collection (ATCC, Cat# CRL-1586). Cells were maintained in Dulbecco’s modified Eagle’s medium (DMEM) w/ l-glutamate, sodium pyruvate (Corning) supplemented with 10% fetal bovine serum (FBS), 10 U penicillin per ml, and 10 mg streptomycin per ml. HCT-8 human cells line was obtained from the ATCC (Cat#CCL-24) and maintained in Roswell Park Memorial Institute 1640 medium (Gibco) supplemented with 10% FBS, 10 U penicillin per ml, and 10 mg streptomycin per ml. Cell lines were supplemented with Normocyn (Invivogen, Cat. ant-nr-1) to prevent Mycoplasma contamination.

### Virus strains

SARS-CoV-2, isolate USA-WA1/2020, was initially obtained from BEI Resources (Cat#NR-52281) and further propagated in Vero E6 cells^52^. Human coronavirus OC43 was obtained from the ATCC (Cat#VR-1558) and propagated on HCT-8 cells following ATCC recommendations.

### Microneutralization assays

Microneutralization (MN) assays for antibody characterization were performed as described^52^. Briefly, Vero E6 cells were seeded in a 96-well cell culture plate with complete Dulbecco’s Modified Eagle Medium (cDMEM)(Corning) [Penicillin-streptomycin (Corning), non-essential amino acids (Corning), 10% FBS (Peak)]. The following day, heat-inactivated serum samples were serially diluted three-fold in 1x minimum essential medium with 2% FBS with a final volume of 200 µl. 80 µl of serum dilution was transferred to a new 96-well plate and 600 Tissue Culture Infectious Dose 50 percent per well of SARS-CoV-2 (80 µl/well) and mixed with serum dilution and incubated for 1 h at 37 °C. Then, cDMEM was removed from Vero e6 cells and 120 µl of virus-serum mixture was added to the cells. The cells were incubated at 37 °C for 1 h. Virus-serum mixture was removed from the cells and 100 µl of serum dilutions and 100 µl of 1xMEM with 2% FBS was added to the cells. The cells were incubated for 24 h and then fixed with 10% paraformaldehyde (Polysciences) for 24 h at 4 °C. Following fixation, the cells were washed with phosphate-buffered saline (Corning) with tween-20 (Fisher) (PBST) and permeabilized with 0.1% Triton X-100 (Fisher) for 15 min at room temperature. The cells were washed three times using PBST and blocked with 3% milk in PBST for 1 h at room temperature. Then, the cells were incubated with mouse antibody 1C7 (anti-SARS N antibody, kindly provided by Dr. Moran) at a dilution of 1:1000 in 1% milk in PBST and incubated for 1 h at room temperature. The cells were washed three times with PBST. Then, the cells were incubated with goat anti-mouse IgG-HRP (Abcam, Cat. ab6823) at a dilution of 1:10,000 in 1% milk in PBST and incubated for 1 h at room temperature. The cells were washed three times with PBST and TMBE Elisa peroxidase substrate (Rockland) was added. After 15 min incubation, sulfuric acid 4.0 N (Fisher) was added to stop the reaction and the readout was done using a Synergy H1 plate reader (BioTek) at an OD450.

### Recombinant proteins

The recombinant spike protein and recombinant RBD of SARS-CoV-2 were generated and expressed as previously described in detail^52,53^. In brief, the mammalian cell codon-optimized nucleotide sequence for the soluble version of the spike protein (amino acids 1-1213) including a C-terminal thrombin cleavage site, signal peptide, hexahistidine tag and T4 foldon trimerization domain were cloned into pCAGGS mammalian expression vector. The sequence of the spike protein was additionally modified to remove the polybasic cleavage site and two proline residues introduced to increase protein stability. The nucleotide sequence for the RBD (amino acids 319-541) including a signal peptide was cloned into pCAGGS. RBD mutants were generated in the pCAGGS RBD construct by changing single residues using site-directed mutagenesis. The expression plasmids encoding for the spike of common human coronavirus 229E, OC43, and HKU1 were obtained from the NIH (kindly provided by Kizzmekia Corbett and Barney Graham) and the expression plasmid encoding for SARS-CoV-2 NP was constructed at Mount Sinai. The recombinant proteins were expressed in Expi293F cells (Thermo Fisher) using the ExpiFectamine 293 Transfection Kit (Thermo Fisher) according to the manufacturer’s protocol. Cell supernatant was harvested, and the proteins purified using Ni-NTA Agarose (Qiagen). The proteins were concentrated in Amicon centrifugal units (EMD Milipore) and correct size confirmed by reducing sodium dodecyl sulfate-polyacrylamide gel electrophoreses. The recombinant S1 subunit of HKU1 was purchased from Sino Biological (Cat. 40021-V08H).

### Enzyme-linked immunosorbent assay (ELISA)

Ninety-six-well microtiter plates (Thermo Fisher) were coated with 50 μL recombinant protein (RBD, SARS-CoV-2 full-length spike, SARS-CoV-2 NP, OC43 spike, 229E spike, or HKU1 spike, respectively) at a concentration of 2 µg/mL overnight, 4 °C. The next day, the plates were washed three times with PBS (phosphate-buffered saline; Gibco) containing 0.1% Tween-20 (T-PBS, Fisher Scientific) using an automatic plate washer (BioTek). After washing, the plates were blocked for 1 h at room temperature with 200 µl blocking solution (PBS-T with 3% (w/v) milk powder (American Bio)) per well. The blocking solution was removed and serum samples diluted to a starting concentration of 1:80, serially diluted 1:3 in PBS-T supplemented with 1% (w/v) milk powder and incubated at room temperature for 2 h. The plates were washed three times with PBS-T and 50 µl anti-human IgG (Fab-specific) horseradish peroxidase antibody (HRP, Sigma, Cat. A0293) diluted 1:3,000 in PBS-T containing 1% milk powder was added to all wells and incubated for 1 h at room temperature. The plates were washed three times using the plate washer and 100 μL SigmaFast o-phenylenediamine dihydrochloride (Sigma) was added to all wells for 10 min. The enzymatic reaction was stopped with 50 μL 3 M hydrochloric acid (Thermo Fisher) per well and the plates read at a wavelength of 490 nm with a plate reader (BioTek). The results were recorded in Microsoft Excel and AUC values were computed by plotting normalized OD values against the reciprocal serum sample dilutions for ELISAs in GraphPad Prism.

### Hemagglutination inhibition (HAI) assay

Serum samples were incubated overnight with receptor-destroying enzyme (RDE; Denka Seiken) for 16–18 h in a 37 °C water bath. Three volumes (relative to serum) of 2.5% sodium citrate solution were added and the resulting solution was heat inactivated at 56 °C in a water bath (30 min). Final serum dilutions were adjusted to 1:10 in PBS. OC43 virus was diluted to a final concentration of 8 HA units/50 µL in fluorescent treponemal antibody HA buffer (BD Biosciences). Twofold dilutions of RDE treated serum (25 µL) were incubated with equal amount of the virus at 8 HA units/50 µL (30 min, room temperature). Chicken red blood cells (RBCs) (Lampire Biological) at 0.5% in HA buffer (50 µL) were added and incubated 45 min at 4 °C. The HAI titer was determined by taking the reciprocal dilution of the last well in which serum inhibited the HA of RBCs.

### Viral loads and qRT-PCR

To detect SARS-CoV-2 RNA in nasal swabs, a modified version of the CDC 2019-nCoV real-time RT-qPCR was used. Primers and probes were commercially available (Integrated DNA Technologies, cat. 10006713, RUO Kit). SARS-CoV-2 primer and probe sets consisted of two 2019-nCoV-specific sets (N1, N2). A third primer set was used to detect host cellular RNaseP. Reactions were run using the QuantiFast Pathogen RT-PCR + IC Kit (QIAGEN, cat. 211454). A list of all primers used, including the names and sequences, is shown in Supplementary Table 3. Assays were run using USA/WA-1/2020 SARS-CoV-2 RNA as a positive control (20,000 genome copies per reaction) and nuclease-free water as a non-template control in a 384-well format. Reactions were performed in duplicate using the following cycling conditions on the Roche LightCycler 480 Instrument II (Roche Molecular Systems, 05015243001): 50 °C for 20 min, 95 °C for 1 s, 95 °C for 5 min, followed by 45 cycles of 95 °C for 15 s and 60 °C for 45 s. Limit of detection for SARS-CoV-2 was determined by using a commercially available plasmid control (Integrated DNA Technologies, cat. 10006625).

### Multiple sequences alignment and conservation scores

MSA to determine the spike protein sequence identity among SARS-CoV-2 (NC_045512.2), and the human endemic betacoronaviruses HKU1 (YP_173238) and OC43 (YP_009555241.1), and alphacoronavirus 229E (NP_073551.1) was performed with ClustalW. Conservation patterns and scores of the spike protein of SARS-CoV-2 were determined using the ConSurf server (https://consurf.tau.ac.il/). Briefly, a MSA of 150 homologous sequences was constructed using MAFFT. Position-specific conservation scores were computed using an empirical Bayesian algorithm and divided into a discrete scale of nine grades. The conservation scores were projected onto the SARS-CoV-2 spike protein in the closed state (PDB ID 6VXX) as a reference.

### Quantification and statistical analysis

All immune assay values were log10-transformed to improve linearity. The GMT and 95% confidence intervals (CI 95%) were computed by taking the exponent (log10) of the mean and of the lower and upper limits of the 95% CI of the log10‐transformed titers. Fold rise was calculated as the ratio between days 3, 7 or convalescent antibody value to baseline levels. GMFR was computed by taking the exponent (log10) of the mean fold rise and of the lower and upper limits of the CI 95% of the log10‐transformed titers. Statistical significance was established at *p* < 0.05. All reported *p* values are based on two‐tailed tests. Correlation (Pearson), linear regression, local regression fit-line and related-sample multiple comparison (Friedman’s two-way analysis of variance by ranks, also known as Friedman’s two-way ANOVA, and pairwise comparison adjusted by Bonferroni correction) were performed using IBM SPSS Statistics (version 26).

### Reporting summary

Further information on research design is available in the Nature Research Reporting Summary linked to this article.

## Supplementary information

Supplementary Information

Reporting Summary

## Data Availability

All data are available in the manuscript or the supplementary materials. Source data are provided with this paper. The accession codes for the Structure of the SARS-CoV-2 spike glycoprotein (closed state) EMD: 21452 and PDB: 6VXX. Source data are provided with this paper.

## Source data

Source Data
