# Supplementary material for: Immunological imprinting of the antibody response in COVID-19 patients
Source: Nat Commun. 2021 Jun 18;12:3781. doi: 10.1038/s41467-021-23977-1 (PMC8213790; doi:10.1038/s41467-021-23977-1)
Supplement: Supplementary file 1 — Supplementary Information [file 41467_2021_23977_MOESM1_ESM.pdf]

Supplementary Information for

Immunological Imprinting of the Antibody Response in COVID-19 Patients

by Teresa Aydillo et al.

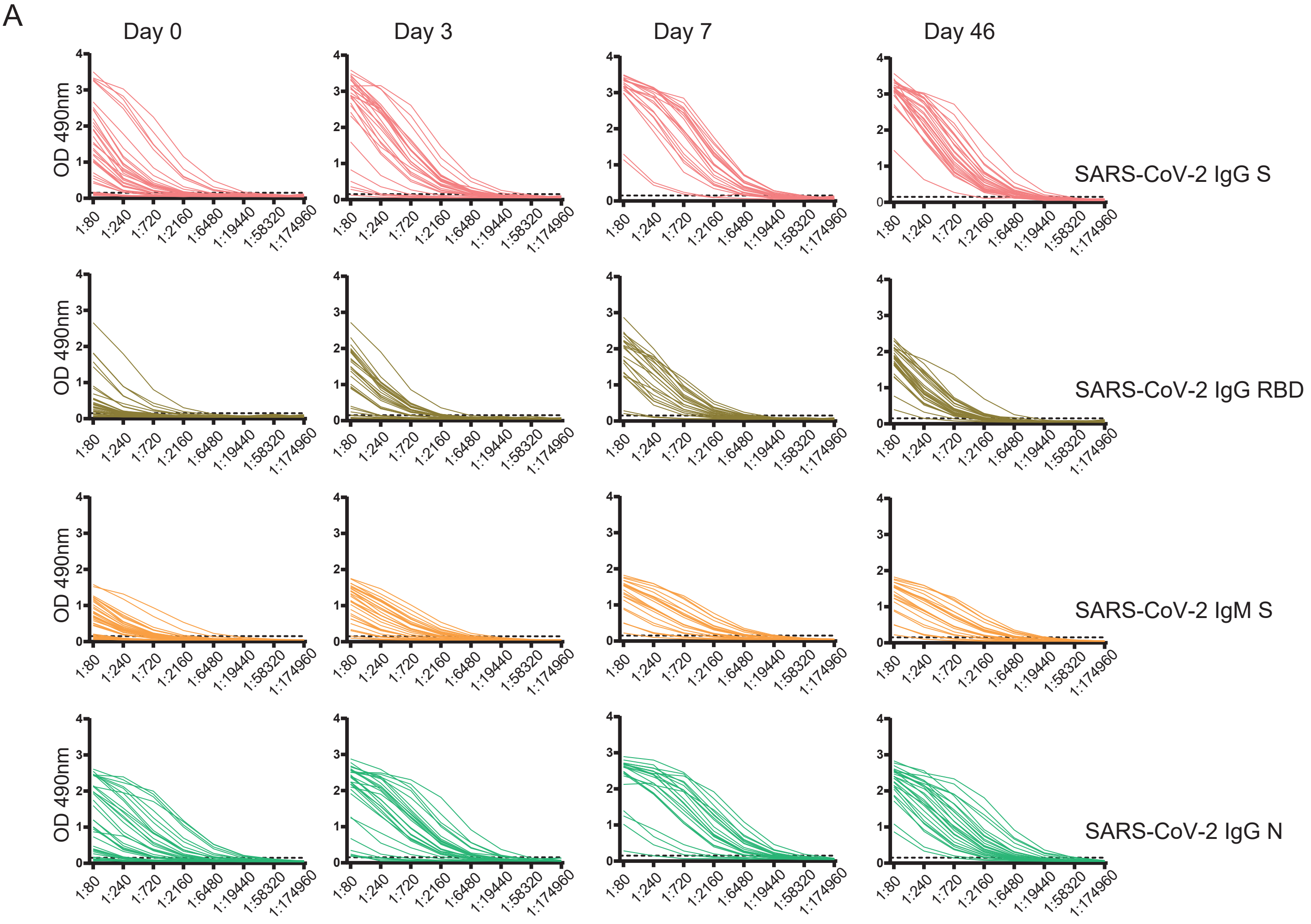

Supplemental Figure 1. Binding profile of the serial dilution curve for each individual serum sample collected at day 0, 3, 7 and 46 in the BACO Cohort. ELISAs assays were performed for the indicated SARS-CoV-2 antigens: IgG spike, IgG RBD, IgM spike and IgG NP. Area-under-the-curve (AUC) values were computed by plotting normalized optical density (OD) values against the reciprocal serum sample dilutions and used to quantify antibody titers for each individual serum.

A

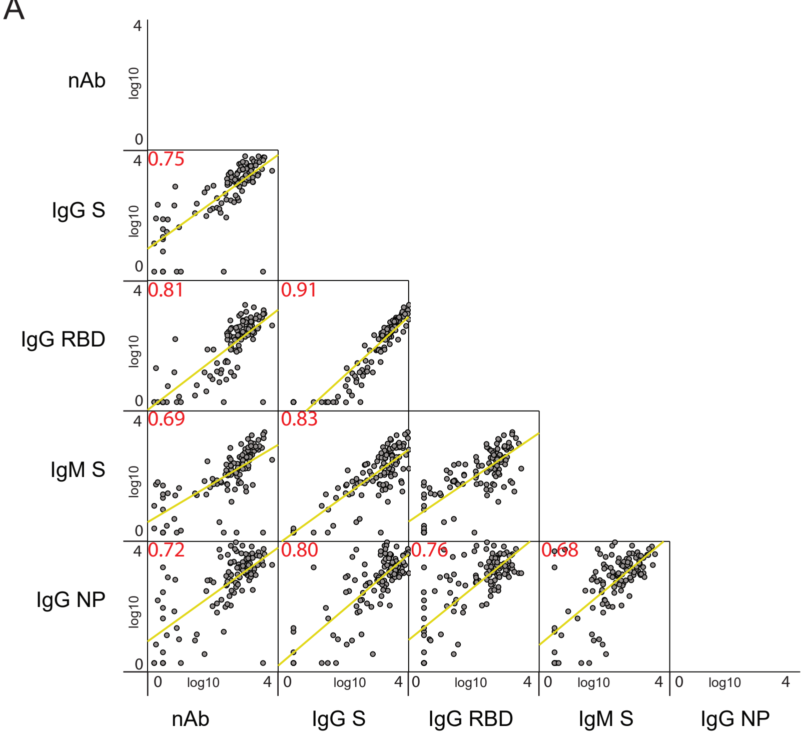

Supplemental Figure 2. Scatterplot matrix of the relationship between measured SARS-CoV-2 antibody responses in the COVID-19 patients. Relationship of each antibody levels is tested for the correlations shown: ELISA AUC titers against SARS-CoV-2 (IgG spike, IgG RBD, IgM spike and IgG NP) and neutralizing titer (IC50%). Pearson coefficient of statistically significant correlations are indicated in red. Fitted linear regression lines for each interaction are shown in yellow. Matrix axis are log10 values scaled from 0 to 4.

A

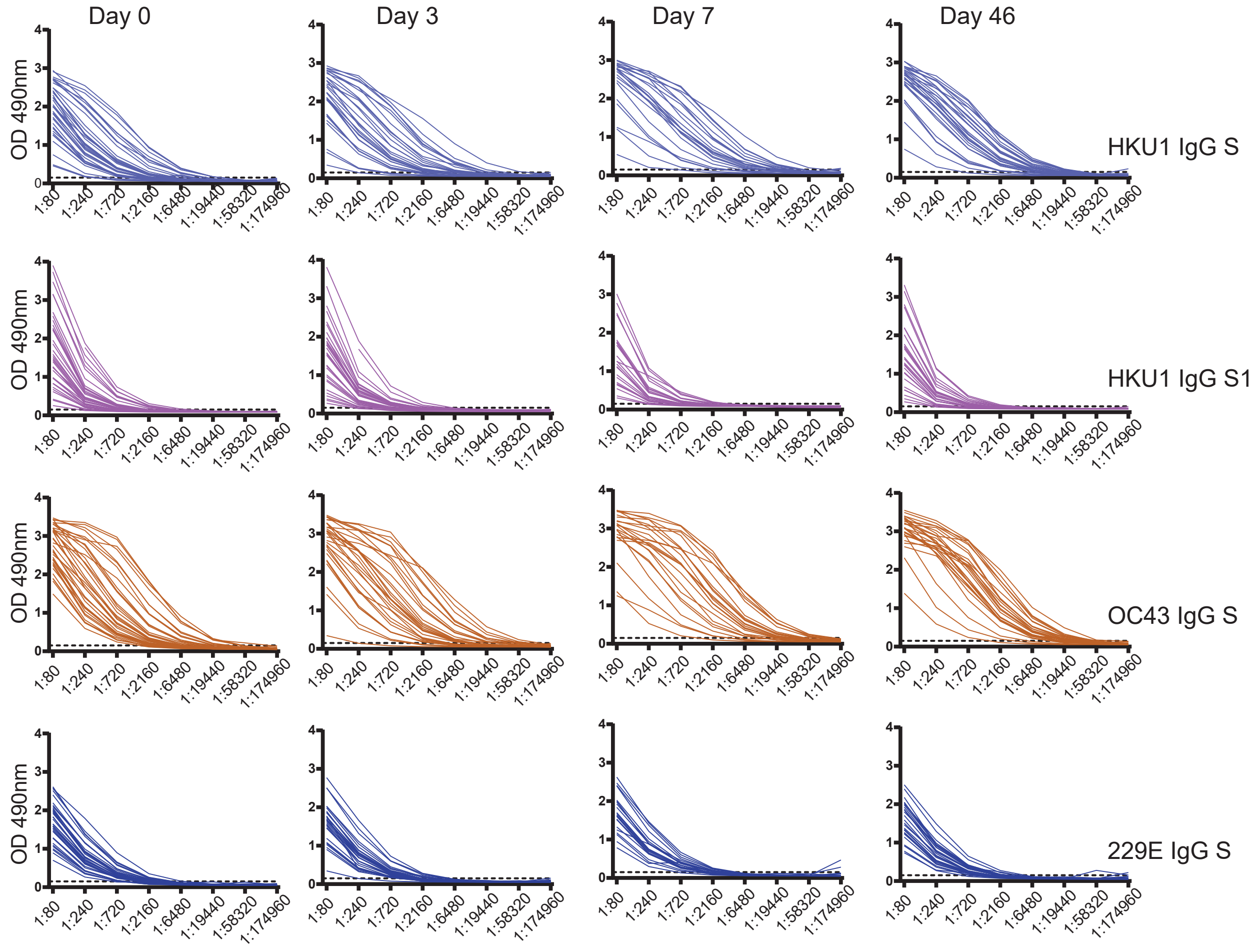

Supplemental Figure 3. Binding profile of the serial dilution curve for each individual serum sample collected at day 0, 3, 7 and 46 in the BACO Cohort. ELISAs were performed for the indicated SARS-CoV-2 antigens: IgG HKU1 spike, IgG HKU1 S1, IgG OC43 spike and IgG 229E. Area-under-the-curve (AUC) values were computed by plotting normalized optical density (OD) values against the reciprocal serum sample dilutions and used to quantify antibody titers for each individual serum.

Supplementary Table 1. Antibody response to the pandemic SARS-CoV-2 and seasonal beta- and alpha-coronaviruses.

	GMT (CI 95%)	GMT (CI 95%)	GMT (CI 95%)	GMT (CI 95%)
	Day 0	Day 3	Day 7	Day 46
SARS-CoV-2 IgG S	134 (53-339)	1161 (490-2749)	5025 (2986-8456)	3552 (2665-4736)
SARS-CoV-2 IgG RBD	12 (6-27)	112 (43-289)	587 (266-1293)	566 (388-825)
SARS-CoV-2 IgM S	47 (22-103)	250 (114-550)	683 (313-1490)	194 (97-389)
SARS-CoV-2 IgG N	150 (53-424)	1187 (467-3017)	3892 (1781-8504)	2305 (1437-3699)
IC50%	58 (19 -174)	201 (52-763)	1373 (735-2565)	601 (330-41095)
HKU1 IgG S	747 (475-1175)	1513 (898-2552)	3363 (1756-6442)	2700 (1763-4134)
HKU1 IgG S1	230 (151-351)	190 (122-294)	146 (87-244)	186 (113-306)
OC43 IgG S	2168 (1460-3220)	3853 (2370-6262)	8027 (4615-13961)	6447 (4471-9295)
HI OC43	75 (50-113)	70 (44-113)	43 (22-85)	99 (62-159)
229E IgG S	455 (356-581)	411 (322-525)	472 (357-624)	373 (286-486)

GMT: geometric mean titer; CI 95%: confidence interval 95%; IgG: immunoglobulin G; IgM: immunoglobulin M; S: spike; RBD: receptor binding domain; IC50%: inhibitory concentration 50%; S1: spike subdomain 1.

Supplementary Table 2. Fold antibodies rise to the pandemic SARS-CoV-2 and seasonal beta- and alpha-coronaviruses.

	GMFR (CI 95%)	GMFR (CI 95%)	GMFR (CI 95%)
	Day 3	Day 7	Day 46
SARS-CoV-2 IgG S	11.6 (6.6-20.2)	44.9 (12.9-155.8)	26.1 (9.6-70.7)
SARS-CoV-2 IgG RBD	12.7 (6.3-25.6)	59.4 (22.1-160.0)	54.8 (24.4-123.3)
SARS-CoV-2 IgM S	7.5 (4.5-12.4)	22.0 (7.2-66.4)	3.8 (1.6-9.0)
SARS-CoV-2 IgG N	12.6 (6.1-26)	32.5 (9.8-107.3)	13.1 (4.7-36.1)
IC50%	6.4 (2.1-19)	23.5 (7.1-78)	11.1 (3.8-31.6)
HKU1 IgG S	2.6 (1.5-2.97)	3.82 (2.4-6.1)	3.1 (2.1-4.5)
HKU1 IgG S1	0.9 (0.8-1.1)	0.8 (0.7-1)	0.8 (0.7-0.9)
OC43 IgG S	2.2 (1.6-3.1)	4 (2.4-6.5)	3 (2.1-4.2)
HI OC43	1 (0.6-1.6)	0.7 (0.3-1.4)	1.4 (1-2)
229E IgG S	1 (0.8-1.2)	0.9 (0.8-1.1)	0.9 (0.7-1)

GMFR: geometric mean fold rise; CI 95%: confidence interval 95%; IgG: immunoglobulin G; IgM: immunoglobulin M; S: spike; RBD: receptor binding domain; IC50%: inhibitory concentration 50%; S1: spike subdomain 1.

Supplementary Table 3. List of primers used to detect SARS-CoV-2 RNA

Name	Description	Oligonucleotide Sequence (5’>3’)
2019-nCoV_N1-F	2019-nCoV_N1 Forward Primer	GAC CCC AAA ATC AGC GAA AT
2019-nCoV_N1-R	2019-nCoV_N1 Reverse Primer	TCT GGT TAC TGC CAG TTG AAT CTG
2019-nCoV_N2-F	2019-nCoV_N2 Forward Primer	TTA CAA ACA TTG GCC GCA AA
2019-nCoV_N2-R	2019-nCoV_N2 Reverse Primer	GCG CGA CAT TCC GAA GAA
RP-F	RNase P Forward Primer	AGA TTT GGA CCT GCG AGC G
RP-R	RNase P Reverse Primer	GAG CGG CTG TCT CCA CAA GT
