# Supplementary material for: Immunological imprinting of the antibody response in COVID-19 patients
Source: Nat Commun. 2021 Jun 18;12:3781. doi: 10.1038/s41467-021-23977-1 (PMC8213790; doi:10.1038/s41467-021-23977-1)
Supplement: Supplementary file 2 — Reporting Summary [file 41467_2021_23977_MOESM2_ESM.pdf]

nature research

Corresponding author(s): Adolfo Garcia-Sastre

Last updated by author(s): May 14, 2021

Reporting Summary

Nature Research wishes to improve the reproducibility of the work that we publish. This form provides structure for consistency and transparency in reporting. For further information on Nature Research policies, see our Editorial Policies and the Editorial Policy Checklist.

Statistics

For all statistical analyses, confirm that the following items are present in the figure legend, table legend, main text, or Methods section.

n/a	Confirmed	
☐	☒	The exact sample size (*n*) for each experimental group/condition, given as a discrete number and unit of measurement
☐	☒	A statement on whether measurements were taken from distinct samples or whether the same sample was measured repeatedly
☐	☒	The statistical test(s) used AND whether they are one- or two-sided<br>*Only common tests should be described solely by name; describe more complex techniques in the Methods section.*
☐	☒	A description of all covariates tested
☐	☒	A description of any assumptions or corrections, such as tests of normality and adjustment for multiple comparisons
☐	☒	A full description of the statistical parameters including central tendency (e.g. means) or other basic estimates (e.g. regression coefficient) AND variation (e.g. standard deviation) or associated estimates of uncertainty (e.g. confidence intervals)
☐	☒	For null hypothesis testing, the test statistic (e.g. *F*, *t*, *r*) with confidence intervals, effect sizes, degrees of freedom and *P* value noted<br>*Give P values as exact values whenever suitable.*
☐	☒	For Bayesian analysis, information on the choice of priors and Markov chain Monte Carlo settings
☒	☐	For hierarchical and complex designs, identification of the appropriate level for tests and full reporting of outcomes
☐	☒	Estimates of effect sizes (e.g. Cohen's *d*, Pearson's *r*), indicating how they were calculated

Our web collection on statistics for biologists contains articles on many of the points above.

Software and code

Policy information about availability of computer code

Data collection	No software was used.
Data analysis	GraphPad Prism (Version 9), IBM SPSS Statistics (version 26), The ConSurf server

For manuscripts utilizing custom algorithms or software that are central to the research but not yet described in published literature, software must be made available to editors and reviewers. We strongly encourage code deposition in a community repository (e.g. GitHub). See the Nature Research guidelines for submitting code & software for further information.

Data

Policy information about availability of data

All manuscripts must include a data availability statement. This statement should provide the following information, where applicable:

- Accession codes, unique identifiers, or web links for publicly available datasets
- A list of figures that have associated raw data
- A description of any restrictions on data availability

All data is available in the manuscript or the supplementary materials. Source data are provided within this paper. The accession codes for the Structure of the SARS-CoV-2 spike glycoprotein (closed state) EMD: 21452 and PDB: 6VXX.

Field-specific reporting

Life sciences study design

All studies must disclose on these points even when the disclosure is negative.

Sample size	An observational prospective human cohort study of subjects with COVID-19 disease was carried out during the first pandemic wave (March-May 2020) of SARS-CoV-2 in Barcelona (Spain) and was termed the BACO Cohort. A positive case was defined according to international guidelines when a nasopharyngeal (NP) swab tested positive for SARS-CoV-2 by reverse transcriptase real-timequantitative polymerase chain reaction (RT-qPCR) upon hospital admission. All patients or their legally authorized representatives that provided informed consent were enrolled. A total of 116 serum were collected in the longitudinal follow up until 2 months to characterize the antibody immune response during the first pandemic wave of SARS-CoV-2. Enrollment was closed in May 2020 when number of cases dropped. No other criteria was used to determine the sample size.
Data exclusions	No data was excluded from the analysis.
Replication	Replication of the sample collection was not performed because the study intended to collect only one serum sample at each time point. ELISA assays Assays were repeated with 8 different substrates. ELISAs for each substrate were run once each. Neutralization assay were performed in duplicates. All attempts at replication were successful.
Randomization	Observational prospective study with no randomization.
Blinding	Blinding of patients was not performed since this study was not interventional, and not comparison between experimental or control groups was not necessary. Performance tests of the ELISA assay was conducted using blinded operators.

Reporting for specific materials, systems and methods

We require information from authors about some types of materials, experimental systems and methods used in many studies. Here, indicate whether each material, system or method listed is relevant to your study. If you are not sure if a list item applies to your research, read the appropriate section before selecting a response.

Materials & experimental systems

n/a	Involved in the study	
☐	☒	Antibodies
☐	☒	Eukaryotic cell lines
☒	☐	Palaeontology and archaeology
☒	☐	Animals and other organisms
☐	☒	Human research participants
☒	☐	Clinical data
☒	☐	Dual use research of concern

Methods

n/a	Involved in the study	
☒	☐	ChIP-seq
☒	☐	Flow cytometry
☒	☐	MRI-based neuroimaging

Antibodies

Antibodies used	1C7 is an unpublished in-house mAb with reactivity to the N protein of SARS-CoV-1 and 2. Anti-human IgG (Fab-specific) horseradish peroxidase antibody (HRP, Sigma, #A0293), goat anti-mouse IgG-HRP (Abcam, Cat. ab6823) are commercially available.
Validation	Secondary antibodies from Sigma and Abcam were validated by the company and tested for specificity. Primary monoclonal antibody 1C7 was validated by binding studies to cells infected with SARS-CoV-2 virus.

Eukaryotic cell lines

Policy information about cell lines

Cell line source(s)	Vero E6, HCT-8 were sourced from ATCC.
Authentication	Cell lines were obtained from a commercial source. After receive, cell were recovered and not further authentication was performed.
Mycoplasma contamination	Cell lines tested negative for Mycoplasma contamination.
Commonly misidentified lines (See ICLAC register)	No commonly misidentified cell lines were used

Human research participants

Policy information about studies involving human research participants

Population characteristics	Patients older than 18 years with SARS-CoV-2 infection. Twenty-five (67.6%) were men.
Recruitment	Patients admitted at the University Hospital of Bellvitge with presence of respiratory illness and laboratory-confirmed SARS-CoV2 infection by reverse-transcription polymerase chain reaction (RT-PCR). All patients or their legally authorized representatives provided informed consent. Patients were recruited upon hospitalization, and therefore imprinting on COVID-19 patients with asymptomatic or very mild infection cannot be assessed.
Ethics oversight	The study protocol was approved by the Institutional Review Board of University Hospital of Bellvitge, Barcelona, Spain; and by the Icahn School of Medicine at Mount Sinai, New York, US.

Note that full information on the approval of the study protocol must also be provided in the manuscript.
